# Dried *Plasmodium falciparum*-infected samples as positive controls for malaria rapid diagnostic tests

**DOI:** 10.1186/1475-2875-11-239

**Published:** 2012-07-23

**Authors:** Michael Aidoo, Jaymin C Patel, John W Barnwell

**Affiliations:** 1 Malaria Branch, Division of Parasitic Diseases and Malaria, Center for Global Health, US Centers for Disease Control and Prevention, 1600 Clifton Road, Atlanta, GA, 30333, USA

## Abstract

**Background:**

Rapid diagnostic tests (RDTs) are central to fulfilling the WHO’s recommendation for parasitologic confirmation of all suspected cases of malaria. RDT performance may be compromised when exposed to the high temperature conditions typical of most malaria endemic regions. However, a systematic method to monitor RDT quality and performance in endemic countries is lacking at the present time. Current methods to monitor RDT performance in the field include comparing results from RDTs to diagnoses made by light microscopy and observing health workers perform tests. These methods are not substitutes for direct quality control. In this study, the suitability of dried *Plasmodium falciparum*-infected blood as quality control samples for malaria RDTs was evaluated.

**Methods:**

Three cultured strains of *P. falciparum* at 200 and 2,000 parasites/μl were tested on 10 brands of RDT. After baseline testing to determine initial reactivity, aliquots of parasite-infected blood were air dried, stored at 35°C, room temperature (~25°C) or 4°C for one, four and 12 weeks and were then tested on the 10 RDTs after rehydration. Extended stability testing of dried blood stored at 4°C was done using *P. falciparum* strain 3D7 at 1,000 and 2,000 parasites/μl.

**Results:**

All dried blood samples at 2,000 parasites/μl retained reactivity (100% sensitivity) at all three temperatures and time points for all nine RDT brands that detect histidine-rich protein-2 (HRP2). The dried blood samples with 200 parasites/μl were detected by six of the nine HRP2-based RDTs at all storage temperatures and time points. The sensitivity for two of the three remaining HRP2-based RDTs was 100% up to four weeks of storage at all temperatures but dropped to 87.5% at week 12. Of the four RDTs that detect plasmodium lactate dehydrogenase (pLDH) in a pan-specific manner, alone or in combination with HRP2, the detection of pLDH in samples with 2,000 parasites/μL was 100% for two RDTs and 80% for the other two RDTs. The mean level for detection of pLDH at 200 parasites/μl was low (29%), with a range of 0% to100%, which was partly attributable to weak initial baseline reactivity. Reactivity of dried 3D7 at 1,000 and 2,000 parasites/μl stored at 4°C was retained at 100% for up to 52 weeks for both HRP2 and pLDH.

**Conclusions:**

In the absence of native or recombinant positive control antigens, well-standardized *P. falciparum*-infected dried blood samples can be used as positive control samples for monitoring RDT performance, particularly with HRP2-detecting tests.

## Background

Malaria remains a serious health problem for much of the global population. Over 200 million cases of *Plasmodium falciparum* occur annually worldwide for which prompt treatment is required to prevent death, especially in children under the age of five. The burden of malaria is highest in sub-Saharan Africa. However, travellers from malaria non-endemic countries are also at increased risk of severe disease if exposed to this parasite. Accurate diagnosis of malaria is critical to administering appropriate treatment. In the past, due to the high prevalence of malaria among febrile patients and the availability of cheap anti-malarial drugs, malaria was diagnosed on the basis of clinical symptoms, with only a small proportion of cases confirmed with laboratory tests. However, the World Health Organization (WHO) updated malaria treatment guidelines in 2010 to emphasize parasitologic confirmation of all suspected cases [1]. This decision was made for multiple reasons, including recent reductions in the incidence of malaria in many endemic countries [2-5], the spread of parasite resistance requiring a switch to more expensive artemisinin-based combination therapy (ACT) and the need to reduce drug pressure to prevent development and spread of resistance to ACT.

Traditionally, malaria diagnosis has relied on light microscopic examination of stained blood smears. However, the capacity to conduct quality routine malaria microscopy has been low, resulting in little or no use of the laboratory to confirm suspected cases and a mistrust of laboratory test results by clinicians [6,7]. In recent years, malaria rapid diagnostic tests (RDTs) have been developed and shown to be comparable or surpass routine microscopy when the use of quality controlled, well performing test kits [8] is coupled with adequate training and carefully designed bench aids [9-12]. RDTs are simple and can be used by non-laboratory staff in health facilities and by community health workers [10-12]. The performance of quality controlled RDTs and their ease of use by a wide variety of non-laboratory health workers have also, in part, led to the new WHO recommendation on parasitologic confirmation of all suspected cases.

Despite their utility, RDTs have several limitations that need to be considered prior to their routine use. First, RDT products from different manufacturers can differ widely in performance characteristics especially in their ability to identify low parasite density infections, and inter-lot variation among some tests from the same manufacturer have been reported [13]. In addition, RDT performance can be compromised when tests are stored for long periods at high temperatures and humidity typical of most malaria-endemic countries [14]. Ensuring good manufacturing quality is addressed to an extent by the RDT product evaluation [13] and pre-procurement lot testing programmes conducted by the WHO, Foundation for Innovative New Diagnostics (FIND) and US Centers for Disease Control and Prevention (CDC). Data from these programmes are available to national malaria control programmes and are intended to ensure the purchase of quality RDTs from manufacturers. In addition, the WHO provides criteria for selecting RDTs with different sensitivities at low parasite densities that is based on malaria endemicity. However, quality control (QC) of RDTs in the field after delivery remains a major challenge. No systematic method exists to monitor the performance of RDTs at the point of care. Such RDT performance monitoring in the field is essential since tests are affected by storage under conditions not specified by manufacturers. One method of QC employed in the field has been to compare results from RDTs to results from light microscopic examination of stained blood smears from the same clinical specimen. This is not an ideal comparison as the two tests detect parasites differently; RDTs detect parasite antigens by immunochromatographic methods whereas microscopy detects whole parasites. As a result of this difference and because parasite antigens, especially HRP2, persist for several days after parasite clearance, RDT positive tests could be negative by smear microscopy in cases where patients have received anti-malarial treatment within a few days or even weeks of being tested [15,16]. Furthermore, accurate slide reading requires proficient microscopists unavailable in most peripheral health facilities in sub-Saharan Africa [6,17]. The second method of observing how well health workers perform RDTs assures the competence of the health worker only and not the quality of the test since the reactivity of patient samples are unknown. Recombinant antigens are being developed for malaria RDTs [18,19]; however, it is not clear when such antigens will be available for use in the field. A method using dried blood spots on filter paper showed some promise; however, the method was not simple, requiring elution of blood from filter paper without a clear correlation of parasite density in the eluted sample to the density in the original sample [20]. In addition, only one culture-derived parasite was tested on three RDT brands, which according to the WHO/FIND Round 2 RDT product testing report had low performance characteristics. Frozen parasite isolates have been used however; the need for freezing parasites at temperatures below −70°C makes the method impractical for most resource-limited malaria endemic settings.

Millions of malaria RDTs are currently being used in malaria endemic countries without suitable methods for QC at the point of care. While the field waits for recombinant antigens, a simple method of using dried parasitized blood in tubes adapted from a similar method used for HIV rapid tests [21] was developed for malaria rapid tests. Here, an evaluation of the method and how it can be used for malaria RDT QC is reported.

## Methods

### Rapid diagnostic test choice

RDTs were chosen based on performance in Round 2 of WHO/FIND/CDC RDT performance evaluations [13] and their availability for purchase (Table 1). Performance criteria used for test selection was a panel detection score (PDS) at 200 parasite/μl of ≥90 for *Plasmodium falciparum.* PDS for *Plasmodium vivax* was not considered for selection since only *P. falciparum* samples were available for this study. Based on the above criterion, 10 RDTs were selected for this study (Table 1). Four of the tests (RDTs 1, 4, 7 and 9) were specific for *P. falciparum only*; with three detecting HRP2 only and the fourth detecting both HRP2 and *P. falciparum*-specific pLDH. Three tests (RDTs 2, 6 and 10) were combination tests detecting HRP2 and pan- specific pLDH. Two other combination tests (RDTs 3 and 8) detected *P. falciparum* and *P. vivax* by HRP2 and *P. vivax*-specific pLDH respectively. One test (RDT 5) was a combination test that recognized *Plasmodium* species by detecting pan-specific pLDH. The same lot of each RDT kit was used for all experiments with the exception of RDT 1. For this RDT the same lot was used for baseline, week 1 and 4 experiments and a different lot used for week 12 experiments. 

**Table 1:** Rapid diagnostic test type and antigen specificity for the 10 RDTs used in testing dried blood samples

**Product**	Number of bands*	**Parasite specificity**	**Manufacturer**	**Product code**	**Pf PDS# @ 200 parasites/μl**
CareStart™ Malaria HRP2/pLDH Pf test	2	Pf	Access Bio, Inc.	G0181	98
CareStart Malaria HRP2/pLDH (Pf/PAN) COMBO	3	Pf/Pan	Access Bio, Inc.	G0131	97
CareStart™ Malaria HRP2/PLDH (Pf/Pv) COMBO	3	Pf/Pv	Access Bio, Inc.	G0161	90
CareStart Malaria HRP2 (Pf)	2	Pf	Access Bio, Inc.	G0141	99
CareStart Malaria pLDH (PAN)	2	Pan	Access Bio, Inc.	G0111	92
First Response Malaria Ag Combo (PLDH/HRP2)	3	Pan	Premier Medical Corporation Ltd	II6FRC30	100
First Response Malaria Ag HRP2	2	Pf	Premier Medical Corporation Ltd	II3FRC30	100
SD BIOLINE Malaria Ag Pf/Pv (HRP2/pLDH)	3	Pf/Pv	Standard Diagnostics, Inc.	05FK80	96
SD BIOLINE Malaria Ag Pf (HRP2)	2	Pf	Standard Diagnostics, Inc.	05FK50-02-4	97
SD BIOLINE Malaria Ag Pf/Pan (HRP2/pLDH)	3	Pf/Pan	Standard Diagnostics, Inc.	05FK60-02- 3	96

* Number of bands including controls.

# Panel Detection Score from WHO Malaria RDT Product Testing Round 2.

### Dried *Plasmodium falciparum*-infected blood preparation

**Figure 1:**
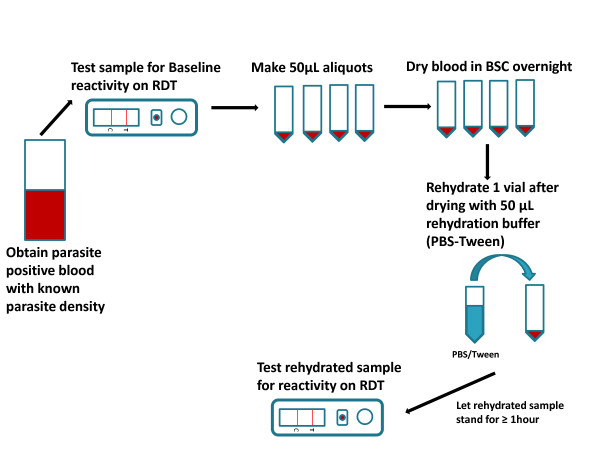
Schematic diagram for preparing dried blood tubes for malaria RDT QC.

Three *in vitro*-cultured *P. falciparum* strains, Nigeria XII, PH 1, and FC27/A3 were used for this study. These parasites were stored as frozen 50 μl aliquots standardized at 200 or 2,000 parasites/μl. Parasites were at ring stage development (>95%) and parasite levels were determined and standardized by established methods [22]. A 50 μl aliquot of frozen blood at 200 and 2,000 parasites/μl for each of the three *P. falciparum* strains was thawed and tested for baseline reactivity. The frozen vials were thawed at room temperature, mixed briefly and tested on each of the 10 RDT brands. Replicate aliquots were then air dried overnight in a bio-safety cabinet. After checking that samples were completely dry, vials were sealed by tightly closing the vial cap and stored in a refrigerator set at 4°C, at room temperature (~25°C) or in a dry incubator set at 35°C. A schematic diagram of dried blood preparation is shown in Figure 1. 

### Dried blood rehydration and rapid diagnostic testing

Dried blood pellets in tubes were rehydrated with PBS-Tween [one sachet of PBS-Tween 20 (SIGMA, USA) dissolved in 1 litre of de-ionized water yielding 0.01 M PBS; 0.138 M NaCl; 0.0027 M KCl and 0.05% Tween 20 at a pH of 7.4]. On the day of testing, one vial of each of the three parasite isolates at 200 and 2,000 parasites/μl and at each storage temperature was rehydrated with 50 μl (same as initial blood volume before drying) of PBS-Tween and left at room temperature for a minimum of 1 hr. Samples were then gently mixed with the use of a micropipette. RDT testing was performed according to the manufacturer’s instructions for each brand. The only exception was the use of a micropipette rather than the test-supplied blood transfer device to transfer 5 μl of blood from the tube onto the test. Samples were tested after one, four and 12 weeks of storage. At each time point, each of the three isolates at 200 and 2,000 parasites/μl was tested for the three storage temperatures (total of 18 tests) except for room temperature-stored Nigeria XII for weeks 4 and 12 and PH1 at 2,000 parasites/μl for all temperatures at week 12 and 35°C at week 4. The former was due to a laboratory error that led to the samples being reconstituted at week 1 and the latter due to unavailability of sufficient number of aliquots. For the purposes of this investigation, a weak reactivity is when a band is distinctly faint when compared to the control band and required brighter light and a second reader to confirm the presence of a band. No densitometry measurements were used to determine band intensity. Band intensity for initial testing of the 10 RDT brands was scored subjectively. However, a band intensity scale became available for scoring of test bands in the extended stability testing and, therefore, an objective “+” scoring system was used. A false negative test was defined as *P. falciparum*-infected blood identified as positive by a test at baseline (before drying) and appearing as a negative test after drying irrespective of storage temperature. For combination tests, the HRP2 and pLDH bands were considered separately.

### Sensitivity calculation

Sensitivity was calculated using the baseline test result on a particular test before drying as the reference gold standard. Therefore sensitivity for dried blood detection was calculated to be product specific. Furthermore, because storage temperature appeared not to influence test results for the 12-week period, sensitivity of dried blood detection was calculated by combining tests results from tubes stored at the three storage temperatures. For combination tests, sensitivity was calculated separately for the HRP2 (Pf) and pLDH (Pan) bands, independently for each product that detected these antigens.

### Dried blood stability

To determine the stability of dried blood over time, a panel of samples using *P. falciparum* clone 3D7 at 1,000 and 2,000 parasites/μl was produced. Cultured 3D7 containing rings or early trophozoite forms were produced using standard malaria culture procedures. A Giemsa-stained smear read by three microscopists was used to determine parasitaemia calculated by the number of infected cells per 1,000 red blood cells. A Coulter Counter® (Beckman-Coulter, Brea, California, USA) was used to determine the number of erythrocytes/μl and the number of parasites/μl subsequently determined from the total number of RBCs/μl. Uninfected blood with a similar haematocrit was used to dilute the cultured parasites to 1,000 and 2,000 parasites/μl. The parasite dilutions obtained were then tested on RDTs 2, 5 and 10 to determine baseline reactivity and then distributed into 60 μl aliquots in tubes, dried as described above and stored at 4°C. At weeks 12, 21, 26, 35, 38, 41, and 52 following dried-tube preparation, vials at 1,000 and 2,000 parasites/μl were rehydrated with 60 μl PBS-Tween and tested on RDT 2 for up to 52 weeks and RDT 5 for up to 46 weeks. Testing was also done on RDT 10 for up to 26 weeks for samples with 1,000 parasites/μl and up to 21 weeks for samples with 2,000 parasites/μl. RDTs 1, 3 and 7 were also tested only at week 52.

### Time series experiments using punctured RDT pouches

To simulate the ability of dried blood to identify faulty RDTs, an experiment was designed in which two sets each of RDTs 2 and 5 were stored either at room temperature (~25°C) or in a humidified incubator set at 37°C. One set each of RDTs at each storage temperature had two 8 mm holes punctured on each side of the test pouch with the desiccant intact. At 2 hrs, 6 hrs, 30 hrs, 2 weeks and 4 weeks, duplicate RDTs were taken from each set and tested using reconstituted dried blood (3D7) at 1000 parasites/μl. Punctured tests stored at room temperature for both RDT2 and RDT5 were not tested at weeks 2 and 4 due to unavailability of tests.

## Results

### Rapid diagnostic test brand-specific reactivity

Overall, four of the 10 RDT brands (RDTs 1, 3, 4, and 8), all HRP2-specific tests, maintained reactivity that was comparable to baseline with dried blood specimens at both parasite concentrations of 200 and 2,000 parasites/μl held at all storage temperatures and time points tested (Additional file 1: Supplementary information a, c, d, h). Two RDT brands (RDT 7 and 9), both HRP2 tests, also maintained reactivity for all samples at both parasite concentrations at all temperatures and time points with the exception of specimens of the PH 1 strain at 200 parasites/μl when stored at 35°C for 12 weeks (Additional file 1: Supplementary information g and i). The remaining four RDT brands (RDT 2, 5, 6 and 10; Additional file 1: Supplementary information b, e, f, j), which lost reactivity in 53 of 506 tests (10.5% false negative), shared certain common characteristics. First, all four tests were combination tests designed to detect *P. falciparum* and the other three human malaria species. Second, with the exception of Nigeria XII parasites at 2,000 parasites/μl (week 1, RT; week 4, 35°C and week 12, 35°C) that were non-reactive with pLDH on RDT 6 (Additional file 1: Supplementary information f), reactivity was lost only for samples at 200 parasites/μl. In addition, for three of four RDTs that detected *P. falciparum* separately from other plasmodium parasites (HRP2 and pLDH as separate bands), reactivity was lost for pLDH and not HRP2 for 2 RDTs (RDTs 2 and 10) at the low (200 parasites/μl) parasite density. However, three samples of PH 1 at 200 parasites/μl were non-reactive for HRP2 on RDT 6, (Additional file 1: Supplementary information f). Finally, weak baseline reactivity of the pLDH band was the single most predictive factor for loss of reactivity after drying blood. The latter characteristic is particularly exemplified by RDT 5, a pLDH only test, in which weak baseline band intensity for all three parasite strains at 200 parasites/μl was predictive of non-reactivity after drying (Additional file 1: Supplementary information e).

### Effect of storage temperature and duration of storage on dried blood reactivity

Storage temperature did not appear to significantly influence the reactivity of the dried blood. For RDTs 1, 3, 4 and 8, no loss in reactivity compared to baseline was observed. In some instances, indications of storage temperature influencing dried blood reactivity could not be confirmed. Loss of reactivity for one parasite isolate stored at 35°C was not associated with a loss of reactivity for another parasite stored at the same temperature and length of storage. For example, RDT 5 at week 1, using dried PH 1 at 200 parasites/μl was non-reactive for samples stored at 4°C and RT but reactive for the sample stored at 35°C. At week 12, the same sample was non-reactive for samples stored at 4°C and 35°C but reactive for the sample stored at RT. Another example is shown by parasite strain FC27/A3 at 200 parasites/μl on RDT5. At week 4, dried blood specimens stored at 4°C and 35°C were non-reactive while that stored at RT was reactive. Considering the weak baseline reactivity of both PH 1 and FC27/A3 at 200 parasites/μl on RDT 5, a pLDH-specific test, it is likely the loss of reactivity was due to the sample being detected close to the limit of detection of the test and not due to storage temperature. A stronger indication of an influence of storage temperature on dried blood reactivity is given by the reactivity of 35°C-stored PH1 at 200 parasites/μl at week 12 on RDTs 7 and 9, both HRP2-specific tests. This was the only non-reactive sample of all parasite/storage temperature/time combinations for these two RDTs.

### Effect of parasite isolate on dried blood reactivity

At comparable parasite densities, similarly stored Nigeria XII and FC27/A3 but not PH 1 retained their reactivity on eight of 10 RDTs (RDTs 1, 3, 4, 5, 7, 8, 9 and 10); implying a parasite-specific reactivity profile. Indeed, at 200 parasites/μl, baseline reactivity for PH 1 was weak or negative on RDTs 2, 5, 6 and 10. In addition, at 200 parasites/μl, PH 1 reactivity was weak for all storage temperatures at weeks 1 and 4 and for 4°C and RT at week 12 on RDT9 (Additional file 1: Supplementary information i). On RDT 7, weak reactivity was observed for this sample stored at 4°C for week 1 and for RT and 35°C for both weeks 4 and 12. At week 12, reactivity was lost for the sample stored at 35°C. In addition to the reactivity on RDTs 7 and 9, weak reactivity for PH 1 at 200 parasites/μl was observed for several samples on RDTs 2, 3, 4, 5, 6, 8 and 10.

### Sensitivity of dried blood detection

Using the baseline result as the gold standard and combining results from all three parasites and storage conditions, all dried blood at 2,000 parasites/μl retained reactivity (100% sensitivity) on all nine RDT brands that detect HRP2 (Table 2). At 2,000 parasites/μl, sensitivity for pLDH (Pan) for two of four combination tests was 100%. The sensitivities for the two remaining RDTs (RDTs 2 and 6) were lower at 80%.

**Table 2:** Sensitivity of detection for dried blood specimens at different parasite densities and storage times

		**Sensitivity**		
		**Week 1**	**Week 4**	**Week 12**
RDT1	200 parasites /μl	100	100	100
	2,000 parasites /μl	100	100	100
RDT2 Pf (Pan)	200 parasites /μl	100 (22)†	100 (75)†	100 (12.5)†
	2,000 parasites /μl	100 (100)	100 (100)	100 (80)#
RDT3	200 parasites /μl	100	100	100
	2,000 parasites /μl	100	100	100
RDT4	200 parasites /μl	100	100	100
	2,000 parasites /μl	100	100	100
RDT5	200 parasites /μl	55.5‡	12.5‡	37.5
	2,000 parasites /μl	100	100	100
RDT6 Pf (Pan)	200 parasites /μl	89 (67)	100 (33)	75 (0)*
	2,000 parasites /μl	100 (89)	100 (86)	100 (80)
RDT7	200 parasites /μl	100	100	87.5#
	2,000 parasites /μl	100	100	100
RDT8	200 parasites /μl	100	100	100
	2,000 parasites /μl	100	100	100
RDT9	200 parasites /μl	100	100	87.5#
	2,000 parasites /μl	100	100	100
RDT10 Pf (Pan)	200 parasites /μl	100 (0 )*	100 (0 )*	100 (33)
	2,000 parasites /μl	100 (100)	100 (100)	100 (100)

† Pan band was weakly reactive for two of three parasites at baseline.

‡ Weakly reactive for all three parasites at baseline.

* Pan test negative for two of three parasites and weakly reactive for the one positive parasite at baseline.

# Reactivity lost for one sample stored at 35°C.

At 200 parasites/μl, dried blood stored at all temperatures and time points were detected at 100% sensitivity on six of nine RDTs for HRP2. The sensitivity on two of the three remaining HRP2 detecting RDTs was 100% up to four weeks of storage at all temperatures, dropping to 87.5% for week 12 samples stored at 35°C. The mean sensitivity of dried blood detection for pLDH was low (29%, range 0-100%) at 200 parasites/μl. This low sensitivity was partly attributable to weak baseline reactivity on the pLDH bands on specific RDT brands. The experiments in this study were not designed to detect false positivity. Nevertheless, for the two RDTs specific for both *P. falciparum* and *P. vivax*, irrespective of storage condition, parasite density and length of storage, in no instance was a *P. vivax* band reactive.

### Dried blood stability

Dried 3D7 at 1,000 and 2,000 parasites/μl stored at 4°C remained reactive after several weeks of storage (Table 3a). RDT 2 (HRP2/pLDH) was reactive for both parasite densities for up to 52 weeks irrespective of the target antigen, although as expected, test band intensities for HRP2 were higher than for pLDH. Because of the unavailability of additional tests, RDT 5 (pLDH) was tested for up to 46 weeks and RDT 10 (HRP2/pLDH) up to 26 weeks for samples at 1,000 parasites/μl and up to 21 weeks for samples at 2,000 parasites/μl. However, at week 52, additional RDT products (RDTs 1, 3 and 7) were positive for both parasite densities (Table 3b).

**Table 3:** **Extended stability results for dried *Plasmodium falciparum *3D7 infected blood specimens**

**a)**									
	**3D7 PD**	**RDT 5**		**RDT 2**			**RDT 10**		
		**C**	**T**	**C**	**Pan**	**Pf**	**C**	**Pan**	**Pf**
Baseline	1000p/Î¼l	++++	++++	++++	++++	++++	++++	++	++++
Week 12		++++	++	++++	++	++++	++++	+	+++
Week 21		++++	++	++++	++	++++	++++	+	+++
Week 26		++++	++	++++	++	++++	++++	+	+++
Week 35		++++	++	++++	++	++++	**ND**		
Week 38		++++	++	++++	++	+++	**ND**		
Week 41		++++	++	++++	++	+++	**ND**		
Week 46		++++	++	++++	++	+++	**ND**		
Week 52		**ND**		++++	++	+++	**ND**		
		**C**	**T**	**C**	**Pan**	**Pf**	**C**	**Pan**	**Pf**
Baseline	2000p/Î¼l	++++	++++	++++	++++	++++	++++	++++	++++
Week 12		++++	++++	++++	+++	++++	++++	++	++++
Week 21		++++	++++	++++	++	++++	++++	++	+++
Week 26		++++	++++	++++	++	+++	**ND**		
Week 35		++++	++++	++++	++	+++	**ND**		
Week 38		++++	++	++++	++	+++	**ND**		
Week 41		++++	++	++++	++	+++	**ND**		
Week 46		++++	++	++++	++	+++	**ND**		
Week 52		**ND**		++++	++	+++	**ND**		
**b)**	**3D7 PD**	**RDT 1**		**RDT 3**			**RDT 7**		
		**C**	**Pf**	**C**	**PV**	**Pf**	**C**	**T**	
Week 52	1000p/Î¼l	++++	++++	++++	-	+++	++++	+++	
	2000p/Î¼l	++++	++++	++++	-	++++	++++	++++	

### Dried blood can identify faulty RDTs

The ability of dried blood to identify faulty RDTs was demonstrated in the time series punctured RDT pouch tests. In these experiments, intact RDT pouches for both RDT2 and RDT5 retained their reactivity irrespective of whether they were stored at room temperature or 37°C up to the 4 weeks of testing. However, punctured tests stored at 37°C in a humidified incubator retained reactivity up to 30 hrs and by week 2 were failing to show test bands with control bands very faint (additional file 2: Supplementary information).

## Discussion

Malaria RDT performance can be adversely affected by storage at temperatures above the manufacturer’s recommended range. According to manufacturer-provided product specifications, most RDTs require maximum storage temperatures of approximately 30°C above which test performance may be compromised, especially if tests are stored for prolonged periods at such temperatures. In sub-Saharan Africa, where RDTs are increasingly being used for malaria diagnosis, ambient temperatures are frequently above 30°C and, therefore, in peripheral health facilities where refrigeration is likely to be unavailable for cool storage of RDTs, high temperature exposure is expected to be common. The length of time a test has to be stored at the higher than the recommended storage temperatures before performance is affected is variable. Data from the WHO/FIND/CDC product evaluation indicate [12] most RDTs retain performance after prolonged periods above required storage temperatures while some others deteriorate and a minority improve in performance. It is not clear what level of heat exposure and for how long a test needs to be exposed for a definitive adverse effect on performance. Therefore, monitoring RDTs stored under such conditions is critical to an effective QC system. Such test monitoring could be achieved by positive controls that are regularly used to test for RDT performance. Unfortunately, because malaria RDTs require fresh or frozen parasitized blood and recombinantly produced positive control antigens are as yet unavailable, field monitoring has largely been ignored as a component of a comprehensive RDT quality assurance system. This is despite the importance of guaranteeing the quality of RDTs especially in peripheral health facilities where they are more likely to be used. In this study, parasite-infected blood dried in tubes was used as a simple method for preserving samples for QC of RDTs. The dried tube method has been successfully used for preserving samples for HIV antibody detection and was slightly modified to detect *P. falciparum* antigens in whole blood.

The use of dried *P. falciparum*-infected blood as QC samples appeared to be a feasible method for monitoring RDT performance. However, these results revealed that, for country-specific programmes standardization of the sample on the specific RDT to be monitored is critical to obtaining desired results. This test-specific standardization will not be desirable for product evaluation or when several products are being compared, since one standard will be required for all RDT products. While dried blood retained reactivity on four RDT brands irrespective of parasite isolate, parasite density, storage temperature and length of time stored, reactivity was lost on other RDTs dependent on these variables. Of note, detection of rehydrated dried blood by HRP2-specific tests or HRP2-specific test lines on combination tests was more consistent compared to pLDH bands. This result is similar to observations reported in the WHO/FIND/CDC RDT product evaluation that shows HRP2 tests to be more sensitive than pLDH. Essentially, the reactivity of the rehydrated dried blood was test-dependent. For instance, at 200 parasites/μl, all three parasite isolates showed weak reactivity at baseline on RDT 5, a pLDH only test, while pLDH reactivity was lost for two of the three parasites on RDTs 6 and 10, both combination tests detecting HRP2 and pLDH. In addition to test-dependent differences, the parasite isolate also appeared to influence reactivity. At 200 parasites/μl, PH 1 was likely being recognized close to its detection limit, which is conferred by the amount of HRP2 it produces. Irrespective of storage temperature and length of storage, PH 1 at this parasite concentration was associated with more weak or lost reactivity than the other parasite isolates irrespective of whether HRP2 or pLDH was the target antigen.

Based on the parasite and test-dependent reactivity patterns, the use of dried blood requires careful characterization and standardization of the parasite isolate for the type of tests to be monitored. This should include the use of standard protocols for generation and dilution of validated and quality controlled samples. Strong baseline reactivity at a required parasite density may be critical to retaining dried blood reactivity after rehydration since weak baseline reactivity may suggest reactivity at the limit of detection of the test and slight intra-lot variations may result in loss of reactivity on some tests. Although this study did not identify any clear temperature-related loss of reactivity of dried blood, the loss of reactivity of PH 1 at 200 parasites/μl stored only at 35°C for 12 weeks on RDTs 7 and 9 suggests a possible temperature effect and therefore prolonged storage should be at cool temperatures. This has implications for the practicality of dried blood stored at peripheral health facilities. One solution could be that dried blood is stored for shorter times at peripheral health facilities if refrigeration is unavailable or that supervisors take along during supervisory visits tubes containing dried samples stored at cool temperatures at a higher level health facility. This is supported by the retention of dried 3D7 reactivity for over 12 months. It was also demonstrated that failing tests could be identified using the dried blood. In addition to their utility as QC samples, dried blood panels could be developed into proficiency testing panels for assessing health workers’ ability to perform RDTs and interpret results. Such a use could double as assessing the competency of health workers and test performance at the same time.

## Conclusions

In the absence of recombinant positive control antigens, well-characterized, dried *P. falciparum*-infected blood in tubes can be used as QC samples for monitoring the quality of RDTs. The samples used should be well characterized and standardized for their baseline reactivity on a range of performance acceptable RDTs. Field testing of this methodology is necessary to assess applicability under field conditions.

## Competing interests

The authors declare that they have no competing interests.

## Authors' contributions

MA conceived and designed the study. MA and JP carried out the experiments. MA, JP and JWB analysed the data and wrote the manuscript. All authors read and approved the final manuscript.

## Supplementary Material

Additional file 1 Supplementary information Dried Tube Experiments. Colour-coded results for 10 RDT brands. Results are shown as relative band intensities for control and test bands. (PDF 207 kb) Click here for file

Additional file 2 Supplementary information Punctured RDT Testing. Time series experiments using punctured RDT pouches. Test results for intact and punctured RDTs 2 and 5 stored at room temperature or 37°C and tested over time. (PDF 189 kb) Click here for file
