# Supplementary material for: Dried Plasmodium falciparum-infected samples as positive controls for malaria rapid diagnostic tests
Source: Malar J. 2012 Jul 23;11:239. doi: 10.1186/1475-2875-11-239 (PMC3483274; doi:10.1186/1475-2875-11-239)
Supplement: Additional file 1 Supplementary information — Dried Tube Experiments. Colour-coded results for 10 RDT brands. Results are shown as relative band intensities for control and test bands. (PDF 207 kb) [file 1475-2875-11-239-S1.pdf]

Additional File 1

a) RDT 1

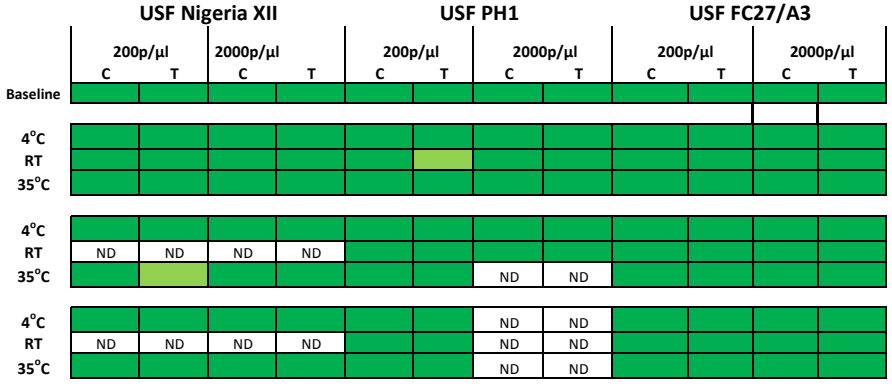

b) RDT 2

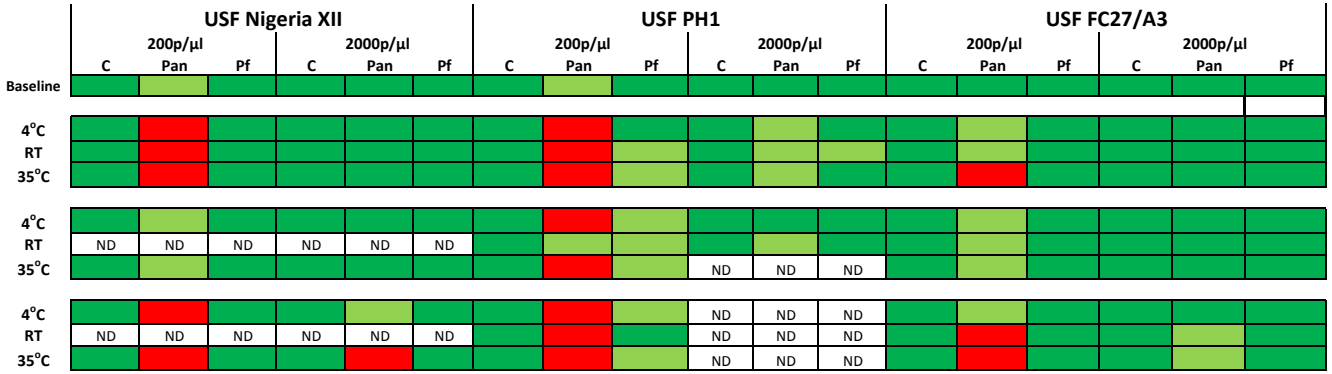

c) RDT 3

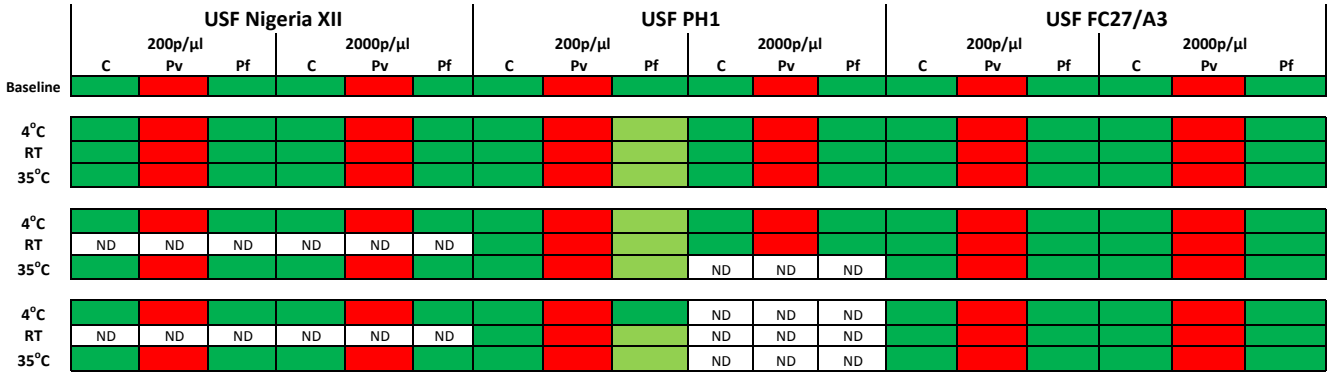

d) RDT 4

		USF Nigeria XII				USF PH1				USF FC27/A3			
		200p/µl		2000p/µl		200p/µl		2000p/µl		200p/µl		2000p/µl	
		C	T	C	T	C	T	C	T	C	T	C	T
	Baseline												
Week 1	4°C												
	RT												
	35°C												
Week 4	4°C												
	RT	ND	ND	ND	ND								
	35°C							ND	ND				
Week 12	4°C							ND	ND				
	RT	ND	ND	ND	ND			ND	ND				
	35°C							ND	ND				

e) RDT 5

		USF Nigeria XII				USF PH1				USF FC27/A3			
		200p/µl		2000p/µl		200p/µl		2000p/µl		200p/µl		2000p/µl	
		C	T	C	T	C	T	C	T	C	T	C	T
	Baseline												
Week 1	4°C												
	RT												
	35°C												
Week 4	4°C												
	RT	ND	ND	ND	ND								
	35°C							ND	ND				
Week 12	4°C							ND	ND				
	RT	ND	ND	ND	ND			ND	ND				
	35°C							ND	ND				

f) RDT 6

		USF Nigeria XII						USF PH1						USF FC27/A3					
		200p/µl			2000p/µl			200p/µl			2000p/µl			200p/µl			2000p/µl		
		C	Pan	Pf	C	Pan	Pf	C	Pan	Pf	C	Pan	Pf	C	Pan	Pf	C	Pan	Pf
	Baseline																		
Week 1	4°C																		
	RT																		
	35°C																		
Week 4	4°C																		
	RT	ND	ND	ND	ND	ND	ND												
	35°C										ND	ND	ND						
Week 12	4°C										ND	ND	ND						
	RT	ND	ND	ND	ND	ND	ND				ND	ND	ND						
	35°C										ND	ND	ND						

g) RDT 7

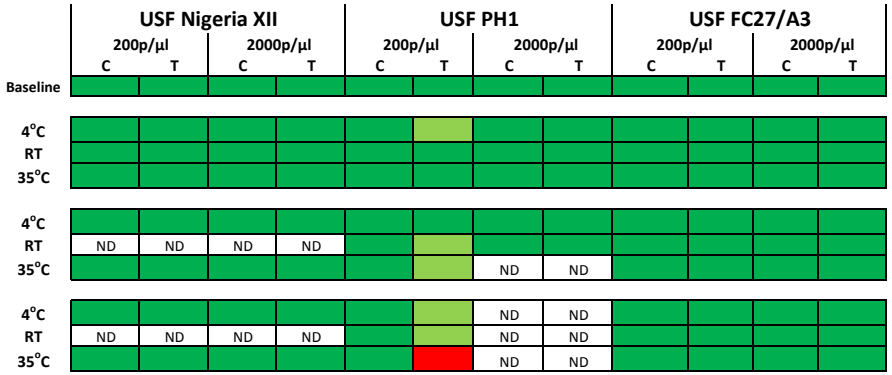

h) RDT 8

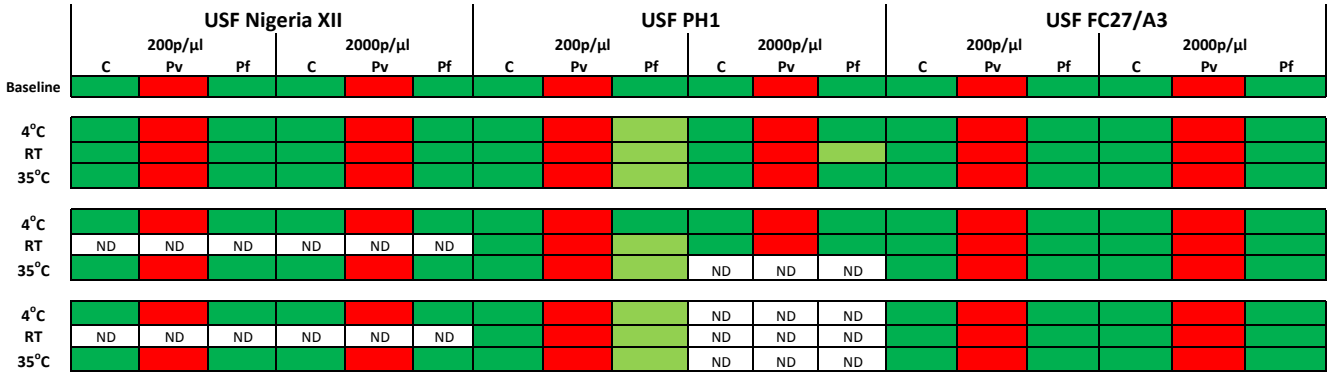

i) RDT 9

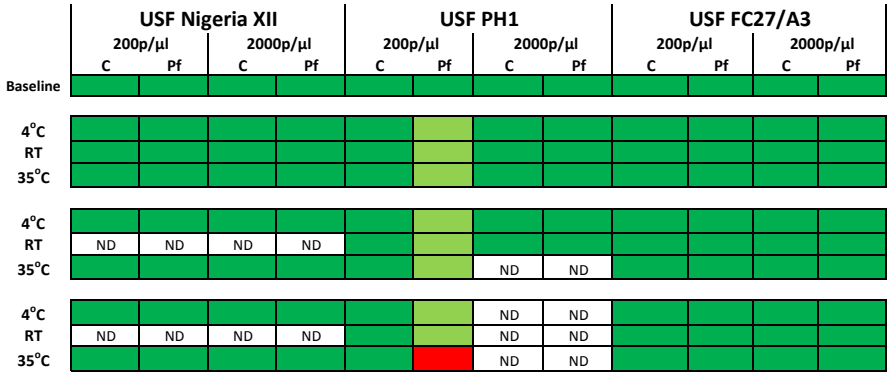

j) RDT 10

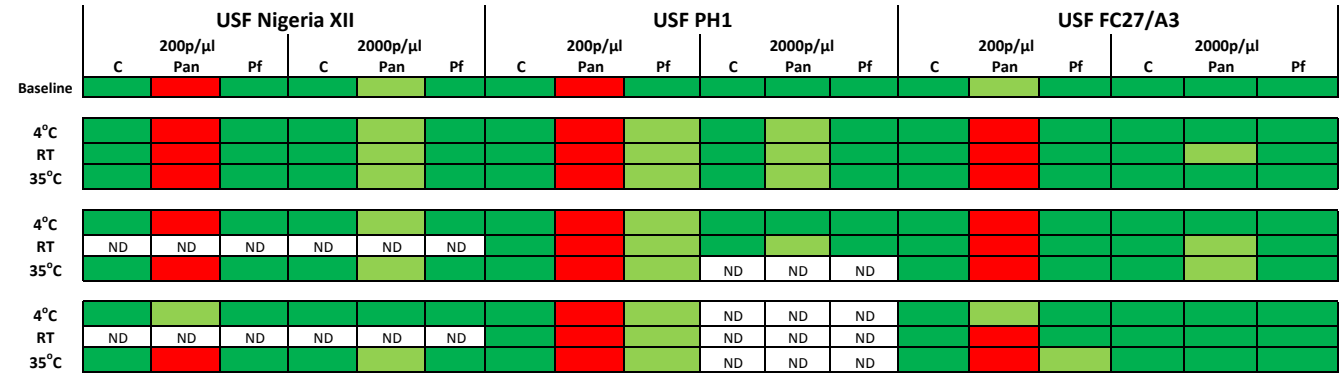

Color Key

- Positive
- Weak Positive
- Negative
