# Supplementary material for: Dried Plasmodium falciparum-infected samples as positive controls for malaria rapid diagnostic tests
Source: Malar J. 2012 Jul 23;11:239. doi: 10.1186/1475-2875-11-239 (PMC3483274; doi:10.1186/1475-2875-11-239)
Supplement: Additional file 2 Supplementary information — Punctured RDT Testing. Time series experiments using punctured RDT pouches. Test results for intact and punctured RDTs 2 and 5 stored at room temperature or 37°C and tested over time. (PDF 189 kb) [file 1475-2875-11-239-S2.pdf]

Time series experiments using punctured RDT pouches

	Room Temperature						37°C Humidified					
	Punctured			Intact			Punctured			Intact		
RDT 2	C	Pan	Pf	C	Pan	Pf	C	Pan	Pf	C	Pan	Pf
2 hrs Test 1	4+	2+	3+	4+	2+	3+	4+	1+	2+	4+	1+	2+
2 hrs Test 2	4+	2+	3+	4+	2+	3+	4+	1+	2+	4+	1+	2+
6 hrs Test 1	4+	2+	2+	4+	2+	2+	4+	1+	2+	4+	1+	2+
6 hrs Test 2	4+	2+	2+	4+	2+	2+	4+	1+	2+	4+	1+	2+
30 hrs Test 1	3+	1+	1+	4+	1+	2+	4+	1+	2+	4+	1+	2+
30 hrs Test 2	4+	1+	2+	4+	1+	2+	4+	1+	2+	4+	1+	1+
Week 2 Test 1	ND	ND	ND	4+	2+	2+	±	±	Neg	4+	1+	2+
Week 2 Test 2	ND	ND	ND	4+	2+	3+	±	±	Neg	4+	1+	2+
Week 4 Test 1	ND	ND	ND	4+	1+	2+	±	Neg	Neg	4+	1+	2+
Week 4 Test 2	ND	ND	ND	4+	1+	2+	±	Neg	Neg	4+	1+	2+

	Room Temperature				37°C Humidified			
	Punctured		Intact		Punctured		Intact	
RDT 5	C	T	C	T	C	T	C	T
2 hrs Test 1	4+	2+	4+	2+	4+	2+	4+	2+
2 hrs Test 2	4+	2+	4+	2+	4+	2+	4+	2+
6 hrs Test 1	4+	2+	4+	2+	4+	2+	4+	2+
6 hrs Test 2	4+	4+	4+	2+	4+	2+	4+	2+
30 hrs Test 1	4+	2+	4+	2+	4+	2+	4+	2+
30 hrs Test 2	4+	2+	4+	2+	4+	1+	4+	1+
Week 2 Test 1	ND	ND	4+	2+	±	Neg	4+	2+
Week 2 Test 2	ND	ND	4+	2+	±	±	4+	2+
Week 4 Test 1	ND	ND	4+	2+	±	Neg	4+	2+
Week 4 Test 2	ND	ND	4+	2+	Neg	±	4+	2+

Sample used was dried 3D7 at 1000 parasite/µl. Punctured RDTPouches had two 8mm holes on each side of the pouch

37°C= humidified incubator set at 37°C
